# Proteomics Approach to the Study of Cattle Tick Adaptation to White Tailed Deer

**DOI:** 10.1155/2013/319812

**Published:** 2013-12-02

**Authors:** Marina Popara, Margarita Villar, Lourdes Mateos-Hernández, Isabel G. Fernández de Mera, José de la Fuente

**Affiliations:** ^1^SaBio, Instituto de Investigación en Recursos Cinegéticos IREC-CSIC-UCLM-JCCM, Ronda de Toledo s/n, 13005 Ciudad Real, Spain; ^2^Department of Veterinary Pathobiology, Center for Veterinary Health Sciences, Oklahoma State University, Stillwater, OK 74078, USA

## Abstract

Cattle ticks, *Rhipicephalus (Boophilus) microplus*, are a serious threat to animal health and production. Some ticks feed on a single host species while others such as *R. microplus* infest multiple hosts. White tailed deer (WTD) play a role in the maintenance and expansion of cattle tick populations. However, cattle ticks fed on WTD show lower weight and reproductive performance when compared to ticks fed on cattle, suggesting the existence of host factors that affect tick feeding and reproduction. To elucidate these factors, a proteomics approach was used to characterize tick and host proteins in *R. microplus* ticks fed on cattle and WTD. The results showed that *R. microplus* ticks fed on cattle have overrepresented tick proteins involved in blood digestion and reproduction when compared to ticks fed on WTD, while host proteins were differentially represented in ticks fed on cattle or WTD. Although a direct connection cannot be made between differentially represented tick and host proteins, these results suggested that differentially represented host proteins together with other host factors could be associated with higher *R. microplus* tick feeding and reproduction observed in ticks fed on cattle.

## 1. Introduction

Ticks are ectoparasites that transmit infectious diseases to humans and animals. In particular, cattle ticks, *Rhipicephalus (Boophilus) microplus*, are a serious threat to animal health and production in many regions of the world [1]. Cattle tick infestations are difficult to control and chemical acaricides have been only partially successful [1]. Therefore, other methods are needed to control cattle tick infestations and tick vaccines were developed in the early 1990s as a cost-effective alternative for the control of tick infestations and pathogen infection, reducing the drawbacks associated with chemical acaricides such as selection of acaricide-resistant ticks and contamination of the environment and animal products with chemical residues [2].

Some ticks feed on a single host species while others such as *R. microplus* infest multiple hosts [3, 4]. Tick-host coevolution likely involves genetic traits of both the host and the vector [4]. Although sympatric isolation and adaptation to cattle and deer have been suggested for *R. microplus* in New Caledonia [5], ticks easily adapt to feeding on new host species [6]. The role of wildlife and particularly of white tailed deer (WTD), *Odocoileus virginianus*, in the maintenance of cattle tick populations has been well established [3, 7–10]. In northern Mexico, *R. microplus* ticks can feed on both cattle and WTD sharing the same pastures [3]. However, although *R. microplus* can complete its developmental cycle on WTD, the weight of engorged females, oviposition, and fertility are reduced by 40%, 58%, and 95%, respectively, when compared to ticks fed on cattle [11, 12]. These studies showed that WTD are physiologically suitable hosts for *R. microplus* [11, 12]. However, the factors responsible for the differences in tick feeding and reproduction observed between ticks fed on cattle and WTD are unknown.

The characterization of the factors affecting the differences in tick feeding and reproduction observed between ticks fed on cattle and WTD is important to understand host effect on tick biology and the possibilities for tick control. Herein, we addressed this question by comparing the proteome of *R. microplus* ticks fed on cattle and WTD. The results showed the presence of differentially represented tick and host proteins that could be potentially associated with the differences observed in tick feeding and reproduction between cattle ticks fed on cattle and WTD.

## 2. Materials and Methods

### 2.1. Tick Collection

Adult female *R. microplus* ticks (Susceptible Media Joya strain, CENAPA, Mexico) were collected in previously reported trials after completing feeding on cattle [13] and WTD [14]. Tick infestation, data collection, and analysis were similar in both experiments [13, 14]. Briefly, five crossbred calves and four 4-5-months-old WTD were purchased from estates in Tamaulipas, Mexico, kept tick-free and not treated with any vaccines prior to infestation with 10,000 *R. microplus* larvae per animal in Spring using an animal facility at the University of Tamaulipas where animals were kept in individual pens during tick infestation and collection [13, 14]. The tick colony was maintained on cattle and thus adapted to WTD in this experiment. Tick larvae were used for infestations at 15 days after hatching from eggs. Engorged female ticks were collected, weighted, and analyzed for oviposition and fertility in a similar way for infestations in cattle and WTD [13, 14]. An equal number of ticks randomly collected from each infested host were mixed and stored at −20°C in 70% ethanol until used for protein extraction.

### 2.2. Protein Extraction

Eight ticks from each group were dissected, cuticle removed, pulverized in liquid nitrogen, and homogenized with a glass homogenizer (10 strokes) in 1 mL buffer (10 mM phosphate buffer saline (PBS), pH 7.4) supplemented with 1% SDS and complete miniprotease inhibitor cocktail (Roche, Basel, Switzerland) per 50 *μ*g sample. Samples were sonicated for 1 min in an ultrasonic cooled bath followed by 10 sec of vortex. After 3 cycles of sonication-vortex, the homogenates were centrifuged at 200 ×g for 5 min at room temperature to remove cellular debris. The supernatants were collected and protein concentration was determined using the BCA Protein Assay (Thermo Scientific, San Jose, CA, USA) using BSA as standard.

### 2.3. Proteomics

Protein extracts (200 *μ*g from each sample) were precipitated following the methanol/chloroform procedure [15], resuspended in 100 *μ*L Laemmli sample buffer, and applied onto 1.2 cm wide wells on a 12% SDS-PAGE. The electrophoresis was stopped as soon as the front entered 3 mm into the resolving gel, so that the whole proteome became concentrated in the stacking/resolving gel interface. The unseparated protein bands were visualized by staining with GelCode Blue Stain Reagent (Thermo Scientific), excised, cut into 2 × 2 mm cubes, and digested overnight at 37°C with 60 ng/*μ*L sequencing grade trypsin (Promega, Madison, WI, USA) at 5 : 1 protein : trypsin (w/w) ratio in 50 mM ammonium bicarbonate, pH 8.8 containing 10% (v/v) acetonitrile [16]. The resulting tryptic peptides from each band were extracted by 30 min-incubation in 12 mM ammonium bicarbonate, pH 8.8. Trifluoroacetic acid was added to a final concentration of 1% and the peptides were finally desalted onto OMIX Pipette tips C_18_ (Agilent Technologies, Santa Clara, CA, USA), dried down, and stored at −20°C until mass spectrometry analysis.

The desalted protein digest was resuspended in 0.1% formic acid and analyzed by RP-LC-MS/MS using an Agilent 1100 LC system (Agilent Technologies) coupled to a linear ion trap LTQ-Velos mass spectrometer (Thermo Scientific). The peptides were separated by reverse phase chromatography using a 0.18 mm × 150 mm Bio-Basic C_18_ RP column (Thermo Scientific) at 1.8 *μ*L/min. Peptides were eluted using a 120 min gradient from 5 to 40% solvent B in solvent A (solvent A: 0.1% formic acid in water; solvent *B*: 0.1% formic acid and 80% acetonitrile in water). ESI ionization was done using a microspray metal needle kit (Thermo Scientific) interface. Peptides were detected in survey scans from 400 to 1600 amu (1 *μ*scan), followed by fifteen data dependent MS/MS scans (Top 15), using an isolation width of 2 mass-to-charge ratio units, normalized collision energy of 35%, and dynamic exclusion applied during 30 sec periods.

### 2.4. Proteomics Data Analysis

Protein identification was carried out using the SEQUEST algorithm (Proteome Discoverer 1.3, Thermo Scientific). The MS/MS raw files were searched against the Ixodida (40,849 entries in June 2013) and Ruminantia (66,519 entries in June 2013) Uniprot databases with the following constraints: tryptic cleavage after Arg and Lys, up to two missed cleavage sites, and tolerances of 1.0 Da for precursor ions and 0.8 Da for MS/MS fragment ions and the searches were performed allowing optional Met oxidation and Cys carbamidomethylation. Due to the limited number of tick proteins included in the database, a false discovery rate (FDR) ≤ 0.05 was considered as condition for successful peptide assignments and subsequent tick protein identification while only host proteins with FDR ≤ 0.01 were considered. Differential protein representation between different samples for tick proteins in blood digestion and reproduction pathways was determined using peptides/protein by *χ* ^2^-test (*P* = 0.05). For host proteins, differential protein representation between different samples was analyzed for individual proteins using *χ* ^2^ statistics with Bonferroni correction in the IDEG6 software (*P* = 0.05) [17]. Two replicates were performed with similar results.

### 2.5. Protein Ontology Assignments

Functional data for each protein were obtained from Uniprot and included gene ontology (GO) annotations, EC number, and Interpro motifs. Assignment of GO terms to identified proteins was done by Blast2GO software (version 2.6.6) in three main steps: blasting to find homologous sequences, mapping to collect GO terms associated with blast hits, and annotation to assign functional terms to query sequences from the pool of GO terms collected in the mapping step [18]. Sequence data of identified proteins were uploaded as FASTA file to the Blast2GO software and the function assignment was based on GO database. The blast step was performed against NCBI public databases through blastp. Other parameters were kept at default values: *e*-value threshold of 1*e*-3, recovery of 20 hits per sequence, minimal alignment length (hsp filter) of 33 (to avoid hits with matching region smaller than 100 nucleotides), and blast mode was set to QBlast-NCBI. Configuration for annotation was an *e*-value-Hit-filter of 1.0*E*-6, annotation cut off of 55, and GO weight of 5. For visualizing the functional information (GO categories: molecular function (MF) and biological process (BP)), the analysis tool of the Blast2GO software was used.

### 2.6. Western Blot Analysis of Cathepsin L

The Western blot analysis of Cathepsin L levels in ticks was performed as previously reported [19]. Briefly, total proteins (150 *μ*g from each sample) were methanol/chloroform precipitated, resuspended in Laemmli sample buffer, and separated on a 15% SDS-PAGE under reducing conditions. After electrophoresis, proteins were transferred to nitrocellulose membranes (Bio-Rad, Hercules, CA, USA), blocked with SuperBlock blocking buffer in TBS (Thermo Scientific), and incubated overnight at 4°C with rabbit polyclonal anti-Cathepsin L (Mature region no. pab0213-0; Covalab, Villeurbanne, France) antibodies. To detect the antigen-bound antibody, membranes were incubated with goat anti-rabbit IgG conjugated with horseradish peroxidase (dilution 1 : 10,000; Sigma-Aldrich, Saint Louis, MO, USA). Immunoreactive proteins were detected by chemoluminescence using the SuperSignal West Pico chemoluminescent substrate (Thermo Scientific), visualized with an ImageQuant 350 Digital Imaging System (GE Healthcare, Pittsburgh, PA, USA), quantified using the ImageQuant TL 7.0 software (GE Healthcare), and normalized against total proteins. Normalized protein levels (*N* = 2) were compared between samples by Student's *t*-test (*P* = 0.05).

## 3. Results 

### 3.1. *R. microplus* Tick Infestations in Cattle and WTD

The same strain of *R. microplus* was used to infest cattle and WTD under similar conditions. Ticks were maintained on cattle until freshly obtained larvae were used to infest cattle and WTD. Thus, ticks were adapted to feed on WTD in this experiment and reduced tick numbers, weight, oviposition, and fertility were obtained in ticks feed on WTD when compared to ticks fed on cattle (Table 1).

### 3.2. Characterization of the Tick Proteome

**Figure 1:**
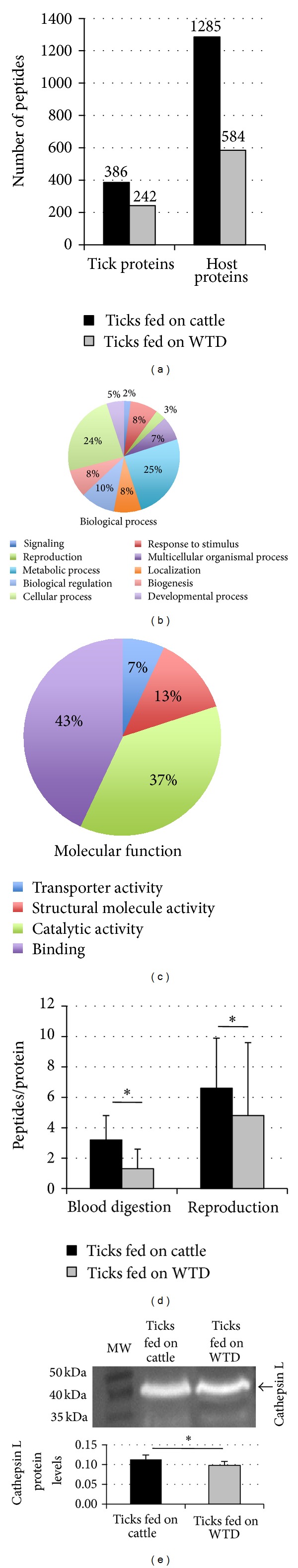
Proteomics characterization of ticks fed on cattle and WTD. (a) Number of peptides for tick and host proteins identified in ticks fed on cattle and WTD. (b) Proteins identified in ticks fed on cattle and WTD were functionally annotated and grouped according to their biological process. (c) Proteins identified in ticks fed on cattle and WTD were functionally annotated and grouped according to their molecular function. (d) Number of peptides for tick protein involved in blood digestion and reproduction identified in ticks fed on cattle and WTD. The number of peptides per protein on each pathway was represented as Ave + S.D. and compared between ticks fed on cattle and WTD by *χ* ^2^ test (*P < 0.05). (e) Cathepsin L protein levels were determined by Western blot in *R. microplus* fed on cattle and WTD, quantified, and normalized against total proteins. Normalized protein levels (Ave + S.D. in arbitrary units) were compared between samples by Student's *t*-test (*P < 0.05; *N* = 2). MW: molecular weight markers.

The proteomics analysis resulted in the identification of 202 and 240 tick and host proteins, respectively, in *R. microplus* fed on cattle and WTD (see Additional file 1 in Supplementary Material available online at http://dx.doi.org/10.1155/2013/319812). The number of peptides used for protein identification was the highest in ticks fed on cattle for both tick and host proteins (Figure 1(a)), pointing at the first difference between ticks fed on cattle and WTD. The GO analysis showed that the most represented BPs corresponded to metabolic (25%) and cellular (24%) processes (Figure 1(b)) while the most represented MFs corresponded to binding (43%) and catalytic activity (37%) (Figure 1(c)). Globally, differences were not observed in the composition of BPs and MFs between ticks fed on cattle and WTD (data not shown). However, the analysis of some pathways such as feeding (blood digestion) and reproduction showed significant differences between ticks fed on cattle and WTD (*P* < 0.05; Figure 1(d)).

### 3.3. Candidate Tick Proteins Associated with Tick Feeding and Reproduction

Tick proteins involved in feeding and reproduction were significantly overrepresented in ticks fed on cattle when compared to ticks fed on WTD (Figure 1(d)) and were selected trying to explain differences in feeding and reproductive performance between ticks fed on cattle and WTD (Table 1). The results of proteomics analysis showed that most of the proteins in these pathways were overrepresented in ticks fed on cattle when compared to ticks fed on WTD (Table 2), a result that was corroborated by Western blot for Cathepsin L (Figure 1(e)). Additionally, tick proteins involved in tick reproduction were also overrepresented in ticks fed on cattle (Figure 1(d) and Table 2).

### 3.4. Candidate Host Proteins Affecting Tick Feeding and Reproduction

Of the 240 host proteins identified in ticks fed on cattle and WTD, significant differences were observed for 11 and 5 proteins overrepresented in ticks fed on WTD and cattle, respectively, (*P* < 0.05; Table 3). Underrepresented tick proteins in blood digestion pathway in ticks fed on WTD (Table 2) correlated with significantly higher levels of the more abundant host blood proteins, Hemoglobin, Haptoglobin, and Albumin in feeding ticks (Table 3). Proteins involved in host immunity such as Alpha-2-macroglobulin, Immunoglobulin-like protein, and Fibrinogen were significantly overrepresented in ticks fed on cattle when compared to ticks fed on WTD (*P* < 0.05; Table 3).

## 4. Discussion

### 4.1. Associations between Differences in Tick Proteome and Tick Feeding and Reproduction

The same strain of *R. microplus* was used to infest cattle and WTD under similar conditions. Ticks were maintained on cattle and adapted to feed on WTD in this experiment showing a reduction in tick numbers, weight, oviposition, and fertility in ticks feed on WTD when compared to ticks fed on cattle (Table 1) similar to those previously reported [8, 9], thus supporting the use of these ticks for the comparative proteomics analysis.

Proteomics analysis showed that tick proteins involved in feeding (blood digestion) and reproduction were overrepresented in ticks fed on cattle when compared to ticks fed on WTD and correlated with significantly higher levels of the more abundant host blood proteins in ticks fed on WTD, suggesting that ticks fed on WTD digested blood poorer than ticks fed on cattle. Blood digestion is critical for tick feeding and reproduction [19, 20], and these parameters were reduced in ticks fed on WTD when compared to ticks fed on cattle (Table 1). Furthermore, failure to properly process Hemoglobin could be toxic for the ticks [20, 21].

The differences observed in tick proteins between ticks fed on cattle and WTD probably reflect differences in host factors potentially associated with tick feeding and reproduction [22, 23]. Ticks fed on cattle were probably ingesting more blood as reflected by higher tick weights (Table 1). Although ticks easily adapt to feeding on new host species [10], host factors such as odor and metabolites could increase blood ingestion in ticks fed on cattle when compared to ticks fed on WTD considering that these ticks were maintained in the laboratory by feeding on cattle [24–27]. Higher levels of host proteins involved in immunity such as Alpha-2-macroglobulin and Immunoglobulin-like proteins in ticks fed on cattle when compared to ticks fed on WTD probably reflected differences in host response to tick infestations [23]. Additionally, host proteins such as Hemoglobin, Immunoglobulin-associated proteins, and Albumin identified herein as differentially represented in ticks fed on cattle or WTD have been potentially associated with host response to tick infestations in cattle [28–33]. However, the exact role of these proteins in tick infestations is unknown [28].

In our experiments, we found significantly higher host Fibrinogen levels in ticks fed on cattle when compared to ticks fed on WTD, providing additional support to the effect of host response to tick infestations. Fibrinogen is an essential component of blood coagulation [34], a process affected by tick feeding through the secretion of proteins that hydrolyze Fibrinogen and delay fibrin clot formation for successful blood pool maintenance and digestion [35, 36]. Additionally, Reck et al. [37] showed that Fibrinogen levels increase in response to *R. microplus* tick infestations in cattle. Therefore, higher Fibrinogen levels in ticks fed on cattle may reflect host response to tick infestations and an indicator of the lower tick infestations observed in WTD. Additionally, the possible role of host Fibrinogen in stimulating the production of Cathepsin and other peptidases in feeding ticks remains to be elucidated as a possible adaptation mechanism to circumvent host responses while promoting a better blood digestion machinery that results in higher tick infestations [38].

### 4.2. Targeting Tick Proteins Involved in Blood Digestion and Reproduction for the Control of Cattle Tick Infestations

The ultimate goal of our research is to develop vaccines for the control of tick infestations and pathogen infection and transmission. The results reported here support the possibility of using tick proteins involved in blood digestion and reproduction identified here as vaccine candidates for the control of cattle tick infestations. Targeting tick proteins that are involved in blood digestion and reproduction will mimic the results observed in ticks fed on WTD with lower tick infestations and reproduction that will ultimately result in control of tick populations.

Vaccines against cattle ticks became available in the early 1990s as a cost-effective alternative for tick control that reduced the use of acaricides and the problems associated with them such as selection of acaricide-resistant ticks, environmental contamination, and contamination of animal products with pesticide residues [2, 39–44]. Currently, only two methods exist for the control of tick infestations in WTD, both involving the use of acaricides [4]. Recently, WTD vaccination with recombinant BM86 and Subolesin tick proteins proved their efficacy for the control of cattle tick infestations [14]. These results showed that deer produced an antibody response that correlated with the reduction in tick infestations similar to results in cattle vaccinated with these antigens [13, 14, 43]. Therefore, tick vaccines appear as an alternative for tick control in cattle and WTD with a possible impact on the transmission of tick-borne pathogens [44].

Recently, cattle vaccination with *R. microplus* Vitellin-degrading cysteine endopeptidase and Yolk cathepsin resulted in the control of cattle tick infestations [45–47]. Additionally, preliminary experiments in sheep vaccinated with *R. microplus* Vitellin, a protein derived from the proteolytic processing of Vitellogenin, showed an effect on the control of cattle tick infestations [48]. These results support considering these proteins for the control of cattle tick infestations and provide additional support for the results shown herein.

## 5. Conclusions

In summary, the results of the proteomics analysis showed that *R. microplus* ticks fed on cattle have overrepresented proteins involved in feeding (blood digestion) and reproduction when compared to ticks fed on WTD. Some of the most abundant host proteins were overrepresented in ticks fed on WTD, correlating with poorer blood digestion machinery. Furthermore, host proteins involved in immunity and other processes were overrepresented in ticks fed on cattle. Although a direct connection cannot be made between differentially represented tick and host proteins, these results suggested that differentially represented host proteins could be associated with overrepresented tick proteins involved in feeding and reproduction in ticks fed on cattle and the lower *R. microplus* tick feeding and reproduction observed in ticks fed on WTD when compared to ticks fed on cattle. Other host proteins and metabolites not identified in this study could also be factors associated with tick feeding and reproduction and potentially involved in the differences observed between ticks fed on cattle and WTD. Higher Fibrinogen levels in ticks fed on cattle may reflect host response to tick infestations and an indicator of the lower tick infestations observed in WTD with possible implications in host-tick coevolution. Finally, previous results from vaccination trials in cattle suggest the possibility of using these tick proteins for the control of cattle tick infestations and provided additional support for the results presented here. These results suggested the existence of host factors potentially associated with tick feeding and reproduction and new candidate protective antigens for the control of cattle tick infestations.

## Supplementary Material

Table S1: Tick and host proteins identified in *R.* microplus fed on cattle and White-tailed deer.

## Figures and Tables

**Table 1:** *R. microplus* infestations in WTD and cattle.

Experimental group	*R. microplus* (Media Joya strain)			
	No. of ticks	Tick weight (mg)	Oviposition	Fertility
WTD	381 ± 195	226 ± 15	85 ± 8	0.05 ± 0.00
Cattle	841 ± 94	297 ± 19	109 ± 10	0.40 ± 0.00
Cattle/WTD ratio (% reduction in ticks fed on WTD when compared to ticks fed on cattle)	2.2* (55%)	1.3* (24%)	1.3* (24%)	8.3* (88%)

Deer (*N* = 4) and cattle (*N* = 5) were infested with 10,000 *R. microplus* larvae/animal applied individually to each animal in separate cotton cells attached to the back of the animals. Adult female tick number, tick weight (mg), oviposition (egg weight (mg)/tick), and egg fertility (larvae weight/egg weight) were compared by *χ* ^2^-test (tick numbers) or Student's *t*-test with unequal variance (tick weight, oviposition, and fertility) between groups (*P < 0.01). Data were obtained from Canales et al. [13] and Carreón et al. [14] for cattle and WTD, respectively.

**Table 2:** Tick proteins involved in feeding and reproduction.

Uniprot accession no.	Description	Ticks fed on cattle	Ticks fed on WTD
		No. of peptides	
Blood digestion			
J9QJ79	Cathepsin L	1	0
Q7YW74	Cathepsin L-like cysteine proteinase B	2	0
	Ave ± S.D.	2 ± 1*	0 ± 0

Reproduction			
B0F457	Vitellogenin	9	6
L7M551	Putative multicellular organism reproduction	2	0
A8WAA7	Vitellogenin-2	10	12
Q5EG54	Vitellogenin	11	5
G9M4L6	Vitellogenin-B	1	1
I3VGB9	Vitellin-degrading cysteine endopeptidase	2	2
Q56CZ1	Yolk cathepsin	3	1
	Ave ± S.D.	5 ± 4*	4 ± 4

The number of peptides per protein on each pathway was represented as Ave ± S.D. and compared between ticks fed on cattle and WTD by *χ* ^2^ test (*P < 0.05).

**Table 3:** Host proteins differentially represented in fed ticks.

Uniprot accession no.	Description	Ticks fed on cattle	Ticks fed on WTD
		No. of peptides	
Over-represented in ticks fed on WTD			
P21380	Hemoglobin subunit beta	10	28*
P21379	Hemoglobin subunit alpha	8	11*
P01971	Hemoglobin subunit alpha	8	11*
Q4TU70	Hemoglobin subunit alpha	7	13*
P02074	Hemoglobin subunit beta-3	7	18*
P02080	Hemoglobin subunit beta-C	4	9*
B6D985	Haptoglobin	2	29*
B1NLF5	Haptoglobin	3	27*
D2U6Q1	Haptoglobin	4	12*
G3X6K8	Haptoglobin	7	10*
B3VHM9	Albumin	28	30*

Over represented in ticks fed on cattle			
Q7SIH1	Alpha-2-macroglobulin	33*	3
L8IE16	Alpha-2-macroglobulin	28*	2
B0JYP6	IGK Immunoglobulin-like protein	9*	0
Q3T101	IGL Immunoglobulin-like protein	10*	0
A5PJE3	Fibrinogen alpha chain	14*	1

Host proteins identified in ticks fed on cattle and WTD were compared by *χ* ^2^ statistics with Bonferroni correction in the IDEG6 software (*P < 0.05).

## References

[1] Graf J-F, Gogolewski R, Leach-Bing N (2004). Tick control: an industry point of view. *Parasitology*.

[2] de la Fuente J, Rodríguez M, Redondo M (1998). Field studies and cost-effectiveness analysis of vaccination with Gavac against the cattle tick *Boophilus microplus*. *Vaccine*.

[3] Lohmeyer KH, Pound JM, May MA, Kammlah DM, Davey RB (2011). Distribution of *Rhipicephalus* (*Boophilus*) *microplus* and *Rhipicephalus* (*Boophilus*) *annulatus* (Acari: Ixodidae) infestations detected in the United States along the Texas/Mexico border. *Journal of Medical Entomology*.

[4] Balashov I (1992). The characteristics of the ixodid tick-vertebrate animal parasitic system. *Parazitologiia*.

[5] de Meeûs T, Koffi BB, Barré N, de Garine-Wichatitsky M, Chevillon C (2010). Swift sympatric adaptation of a species of cattle tick to a new deer host in New Caledonia. *Infection, Genetics and Evolution*.

[6] Balashov I (1989). The coevolution of ixodid ticks and terrestrial vertebrates. *Parazitologiia*.

[7] Pound JM, George JE, Lohmeyer KH, Davey RB (2010). Evidence for role of white-tailed deer (Artiodactyla: Cervidae) in epizootiology of cattle ticks and southern cattle ticks (Acari: Ixodidae) in reinfestations along the Texas/Mexico border in South Texas: a review and update. *Journal of Economic Entomology*.

[8] Racelis AE, Davey RB, Goolsby JA, de León AAP, Varner K, Duhaime R (2012). Facilitative ecological interactions between invasive species: *Arundo donax * as favorable habitat for cattle ticks (Acari: Ixodidae) along the U.S.-Mexico border. *Journal of Medical Entomology*.

[9] George JE (1990). Wildlife as a constraint to the eradication of *Boophilus* spp (Acari: Ixodidae). *Journal of Agriculture Entomology*.

[10] Kistner TP, Hayes FA (1970). White-tailed deer as hosts of cattle fever-ticks. *Journal of Wildlife Diseases*.

[11] Graham OH, Price MA (1966). Some morphological variations in *Boophilus annulatus microplus* (Acarina: Ixodidae) from northern Mexico. *Annals of the Entomological Society of America*.

[12] De la Vega R (1984). Aspectos del desarrollo de la garrapata del Ganado vacuno (*Boophilus microplus*) sobre el venado de cola blanca (*Odocoileus virginianus*). *Revista de Salud Animal*.

[13] Canales M, Almazán C, Naranjo V, Jongejan F, de la Fuente J (2009). Vaccination with recombinant *Boophilus annulatus* Bm86 ortholog protein, Ba86, protects cattle against *B. annulatus* and *B. microplus* infestations. *BMC Biotechnology*.

[14] Carreón D, de la Lastra JMP, Almazán C (2012). Vaccination with BM86, subolesin and akirin protective antigens for the control of tick infestations in white tailed deer and red deer. *Vaccine*.

[15] Wessel D, Flügge UI (1984). A method for the quantitative recovery of protein in dilute solution in the presence of detergents and lipids. *Analytical Biochemistry*.

[16] Shevchenko A, Tomas H, Havliš J, Olsen JV, Mann M (2007). In-gel digestion for mass spectrometric characterization of proteins and proteomes. *Nature Protocols*.

[17] Bortoluzzi S, d’Alessi F, Romualdi C, Danieli GA (2001). Differential expression of genes coding of ribosomal proteins in different human tissues. *Bioinformatics*.

[18] Conesa A, Götz S, García-Gómez JM, Terol J, Talón M, Robles M (2005). Blast2GO: a universal tool for annotation, visualization and analysis in functional genomics research. *Bioinformatics*.

[19] Popara M, Villar M, Mateos-Hernández L (2013). Lesser protein degradation machinery correlates with higher BM86 tick vaccine efficacy in *Rhipicephalus annulatus* when compared to *R. microplus*. *Vaccine*.

[20] Horn M, Nussbaumerová M, Šanda M (2009). Hemoglobin digestion in blood-feeding ticks: mapping a multipeptidase pathway by functional proteomics. *Chemistry and Biology*.

[21] Sojka D, Franta Z, Horn M, Caffrey CR, Mareš M, Kopáček P (2013). New insights into the machinery of blood digestion by ticks. *Trends in Parasitology*.

[22] Szabó MP, Morelli J, Bechara GH (1995). Cutaneous hypersensitivity induced in dogs and guinea-pigs by extracts of the tick *Rhipicephalus sanguineus* (Acari: Ixodidae). *Experimental and Applied Acarology*.

[23] Szabó MP, Bechara GH (1997). Immunisation of dogs and guinea pigs against *Rhipicephalus sanguineus* ticks using gut extract. *Veterinary Parasitology*.

[24] Harrison A, Robb GN, Bennett NC, Horak IG (2012). Differential feeding success of two paralysis-inducing ticks, *Rhipicephalus warburtoni* and *Ixodes rubicundus* on sympatric small mammal species, *Elephantulus myurus* and *Micaelamys namaquensis*. *Veterinary Parasitology*.

[25] Louly CC, Soares SF, da Nóbrega Silveira D, Guimarães MS, Borges LM (2010). Differences in the behavior of *Rhipicephalus sanguineus* tested against resistant and susceptible dogs. *Experimental and Applied Acarology*.

[26] Donzé G, McMahon C, Guerin PM (2004). Rumen metabolites serve ticks to exploit large mammals. *Journal of Experimental Biology*.

[27] Osterkamp J, Wahl U, Schmalfuss G, Haas W (1999). Host-odour recognition in two tick species is coded in a blend of vertebrate volatiles. *Journal of Comparative Physiology A *.

[28] Neto LRP, Jonsson NN, D’Occhio MJ, Barendse W (2011). Molecular genetic approaches for identifying the basis of variation in resistance to tick infestation in cattle. *Veterinary Parasitology*.

[29] Kongsuwan K, Piper EK, Bagnall NH (2008). Identification of genes involved with tick infestation in *Bos taurus* and *Bos indicus*. *Developments in Biologicals*.

[30] Kongsuwan K, Josh P, Colgrave ML (2010). Activation of several key components of the epidermal differentiation pathway in cattle following infestation with the cattle tick, *Rhipicephalus* (*Boophilus) microplus*. *International Journal for Parasitology*.

[31] Nascimento CS, Machado MA, Guimarães SE (2010). Differential expression of genes in resistant versus susceptible Gyr x Holstein cattle challenged with the tick *Rhipicephalus* (*Boophilus*) *microplus*. *Genetics and Molecular Research*.

[32] Bagnall N, Gough J, Cadogan L, Burns B, Kongsuwan K (2009). Expression of intracellular calcium signalling genes in cattle skin during tick infestation. *Parasite Immunology*.

[33] Piper EK, Jackson LA, Bagnall NH, Kongsuwan KK, Lew AE, Jonsson NN (2008). Gene expression in the skin of *Bos taurus* and *Bos indicus* cattle infested with the cattle tick, *Rhipicephalus* (*Boophilus*) microplus. *Veterinary Immunology and Immunopathology*.

[34] Davie EW, Ratnoff OD (1964). Waterfall sequence for intrinsic blood clotting. *Science*.

[35] Francischetti IM (2010). Platelet aggregation inhibitors from hematophagous animals. *Toxicon*.

[36] Anisuzzaman A, Islam MK, Alim MA (2011). Longistatin, a plasminogen activator, is key to the availability of blood-meals for ixodid ticks. *PLoS Pathogens*.

[37] Reck J, Berger M, Terra RM (2009). Systemic alterations of bovine hemostasis due to *Rhipicephalus* (*Boophilus*) *microplus* infestation. *Research in Veterinary Science*.

[38] Rodriguez-Valle M, Lew-Tabor A, Gondro C (2010). Comparative microarray analysis of *Rhipicephalus* (*Boophilus*) *microplus* expression profiles of larvae pre-attachment and feeding adult female stages on *Bos indicus* and *Bos taurus* cattle. *BMC Genomics*.

[39] de La Fuente J, Kocan KM (2006). Strategies for development of vaccines for control of ixodid tick species. *Parasite Immunology*.

[40] de la Fuente J, Almazán C, Canales M, de la Lastra JMP, Kocan KM, Willadsen P (2007). A ten-year review of commercial vaccine performance for control of tick infestations on cattle. *Animal Health Research Reviews*.

[41] de la Fuente J, Moreno-Cid JA, Canales M (2011). Targeting arthropod subolesin/akirin for the development of a universal vaccine for control of vector infestations and pathogen transmission. *Veterinary Parasitology*.

[42] Willadsen P (2006). Tick control: thoughts on a research agenda. *Veterinary Parasitology*.

[43] Merino O, Almazán C, Canales M (2011). Targeting the tick protective antigen subolesin reduces vector infestations and pathogen infection by *Anaplasma marginale* and *Babesia bigemina*. *Vaccine*.

[44] de la Fuente J (2012). Vaccines for vector control: exciting possibilities for the future. *The Veterinary Journal*.

[45] Leal AT, Seixas A, Pohl PC (2006). Vaccination of bovines with recombinant *Boophilus* Yolk pro-Cathepsin. *Veterinary Immunology and Immunopathology*.

[46] Seixas A, Leal AT, Nascimento-Silva MC, Masuda A, Termignoni C, da Silva Vaz I (2008). Vaccine potential of a tick vitellin-degrading enzyme (VTDCE). *Veterinary Immunology and Immunopathology*.

[47] Parizi LF, Reck J, Oldiges DP (2012). Multi-antigenic vaccine against the cattle tick *Rhipicephalus* (*Boophilus*) *microplus*: a field evaluation. *Vaccine*.

[48] Tellam RL, Kemp D, Riding G (2002). Reduced oviposition of *Boophilus microplus* feeding on sheep vaccinated with vitellin. *Veterinary Parasitology*.

